# Critical Role of Inflammation and Specialized Pro-Resolving Mediators in the Pathogenesis of Atherosclerosis

**DOI:** 10.3390/biomedicines10112829

**Published:** 2022-11-06

**Authors:** Subhapradha Rangarajan, Davit Orujyan, Patrida Rangchaikul, Mohamed M. Radwan

**Affiliations:** 1 College of Osteopathic Medicine of the Pacific, Western University of Health Sciences, Pomona, CA 91766, USA; 2 Department of Translational Research, College of Osteopathic Medicine of the Pacific, Western University of Health Sciences, Pomona, CA 91766, USA

**Keywords:** cardiovascular disease, atherosclerosis, efferocytosis, inflammation, resolution, immunotherapy

## Abstract

Recent research on how the body resolves this inflammation is gaining traction and has shed light on new avenues for future management of cardiovascular diseases. In this narrative review, we discuss the pathophysiological mechanisms of atherosclerosis, the recent development in the understanding of a new class of molecules called Specialized Pro-resolving Mediators (SPMs), and the impact of such findings in the realm of cardiovascular treatment options. We searched the MEDLINE database restricting ourselves to original research articles as much as possible on the complex pathophysiology of atherosclerosis and the role of SPMs. We expect to see further research in translating these findings to bedside clinical trials in treating conditions with a pathophysiological basis of inflammation, such as coronary artery disease, asthma, and periodontal disease.

## 1. Introduction

Cardiovascular diseases have been ranked as one of the top causes of death in the United States since 1980, with over 600,000 deaths recorded in 2018, irrespective of ethnicity [1].

Atherosclerosis, a disease of the arteries set off initially by fat deposition, is a major cause of life-threatening cardiovascular events. It was long thought to be a passive process caused by the accumulation of cholesterol within the lumen of arteries resulting in ischemia and an eventual complete blockage. However, in recent decades studies proved that plaque inflation and rupture are the events that lead to the life-threatening consequences of atherosclerosis [2,3].

Arteries are composed of endothelial cells (EC), elastin, collagen, and smooth muscle cells [4]. ECs line the lumen of vessels and are subject to physical demands, such as shear stress, imposed by the flow of blood. Such stressors fluctuate and vary through the length of the artery, owing to the rheological properties of the blood and vulnerable areas of the arteries, such as branching points. These factors contributed to the initial focus of the pathogenesis of atherosclerosis and amplified through environmental factors such as age-related arterial degeneration, lifestyle choices of diet and exercise, and other risk factors such as obesity, hypertension, hyperlipidemia, diabetes mellitus, and smoking [2,5,6].

Recently the concept of inflammation resolution has garnered attention, as studies showed that the end of acute inflammation is an active concerted effort by a class of molecules termed SPMs and not a passive process that fizzles away in time. Thus, allowing us to look at treating inflammation by enhancing its resolution. Since atherosclerosis is a chronic inflammation that takes years, a temporal dependency dictates the efficacy and efficiency of such processes [7,8]. This temporal dependency poses a challenge in identifying risk groups with current diagnostic tools since not all individuals with the same cholesterol level end up with the same stage of atherosclerosis, and the lumens of such vulnerable arteries are hard to access at times [2].

Current treatments for atherosclerosis include reducing blood cholesterol levels and surgical upkeep of the arterial lumen—based on the knowledge that cholesterol accumulation is the precursor of its pathogenesis; some developments have been made in treating atherosclerosis with anti-inflammatory medications [9].

## 2. Pathogenesis of Atherosclerosis

The stressors discussed above subject the EC to injury. Intact ECs cannot regenerate the nearby wounded site or migrate distally to an injured site to repair, causing the incessant injury to exhaust EC’s turnover capacity. Lack of a repair mechanism results in perpetually dysfunctional ECs. These cells lose their tight junctions and become more permeable to molecules that otherwise would not be able to enter the intima. The ECs undergo alterations in their adhesive characteristics, becoming ‘sticky,’ leading to more monocyte and T cell attachment in early plaque formation and during plaque growth, respectively. Impaired ECs also exhibit growth-stimulatory characteristics, enabling the entry of LDL molecules and monocytes into the intimal layer, triggering the formation of a fatty streak, the first step in the long process of plaque formation [6,10].

Apo B-100 receptors of the LDL particles bind to the proteoglycan molecules of the extracellular matrix, enter the subintimal layer, and get oxidized. While their oxidation process is multifactorial and not fully understood, it is postulated that Nitric Oxide Synthase generated by the activated macrophages might have a significant role in it. Myeloperoxidase, 15-lipooxygenase, hypochlorous acid, and phenoxy radical intermediates also bring about such oxidation [2]. Oxidized molecular species modify the Lysine residues of Apo B 100 [11].

In atherosclerosis, the oxidized LDL (ox-LDL) induces the release of chemical mediators by the cells in its vicinity and promotes the accumulation of macrophages. This process initiates plaque formation and recruits inflammatory cells, beginning the process of chronic inflammation in the arterial wall [11,12].

Within the intima layer, the damaged ECs release macrophage colony-stimulating factor (M-CSF), which converts the initial set of monocytes into macrophages. These, in turn, generate monocyte chemoattractant protein (MCP-1), which increases the number of immunocompetent cells in the region. Studies have shown that MCP−/− and LDLR−/− mice do not have a risk of atherosclerosis [13]. MCP-1 and the ox-LDL particle are the essential chemokines involved in atherosclerosis [3]. In addition, LDL oxidation generates reactive aldehydes and truncated lipids that trigger a pro-inflammatory cascade in ECs and the expression of adhesion molecules such as VCAM-1, E-Selectin, and P-Selectin. Receptors for MCP-1 on monocytes are heavily upregulated during early plaque formation and are expressed by endothelial cells, smooth muscle cells, and macrophages [2,3,5,6]. Transient blockage of P-selectin, one of the receptors on EC or its ligand, in an apoe−/−, cholesterol-fed mice before the incident of vascular injury resulted in a substantial reduction of neointima formation [14].

Macrophages phagocytose ox-LDL molecules through the scavenger receptors SR-A and CD36. An increase in ox-LDL intake does not downregulate these receptors, so macrophages can potentially keep intaking these particles until they undergo apoptosis [2]. In healthier conditions or with high HDL presence, such macrophages can transfer the LDL species to the HDL molecules to be circulated back to the liver. Apoptosis of macrophages spills out the ingested ox-LDL giving it the color and the name fatty streak [2,3,5,6].

Vascular Smooth Muscle Cells (VSMC) are called into the growing plaque within the intima and generate collagen, elastin, and other extracellular proteoglycans that give the plaque its fibrous cap [5,6]. Even as the fibrous cap provides uniformity and stability to the plaque, at its core is a soft lipid and necrotic material that, with enough growth, occludes the lumen. On the other hand, if this lesion is not uniform, its stability is compromised, allowing the possibility of rupture from shear stress [6].

## 3. Plaque Stability

**Figure 1:**
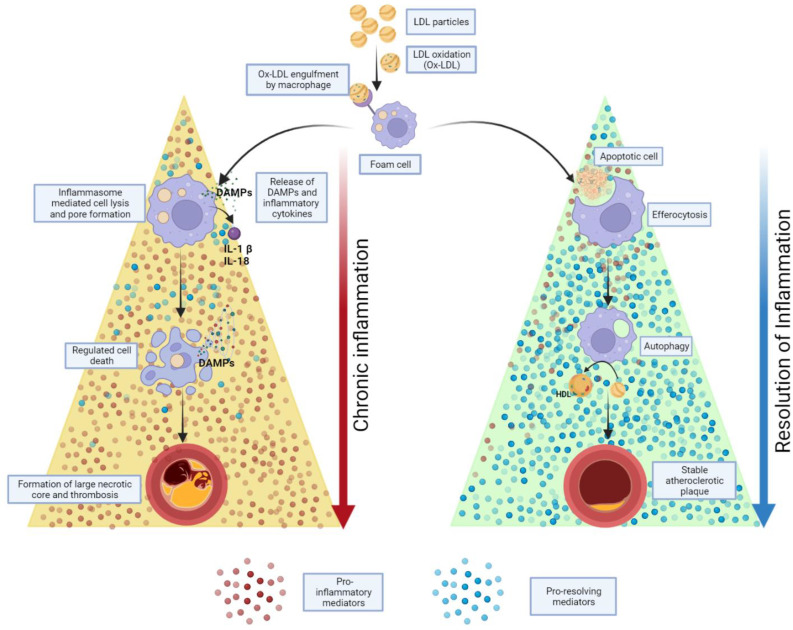
The proportion of pro-inflammatory and pro-resolving mediators in its microenvironment decides the stability and fate of a growing plaque. An abundance of pro-inflammatory mediators, results in inflammasome mediated pore formation leading to inflamed cell death and further release of proinflammatory mediators such as DAMPs, prolonging the inflammation cycle and rendering the plaque unstable and prone to thrombus formation. On the contrary, with an abundance of SPMs in the milieu leads to efferocytosis, a non-phlogistic clearance of any cellular debris, and regulated autophagy leading to a stable plaque.

It has been established that it is the “stability” of the plaque rather than its formation, the culprit behind life-threatening cardiovascular events (Figure 1). The process in which a “fatty streak” morphs into an unstable plaque that ruptures, involves a complex interplay of biochemicals between subsets of different types of immune-modulating cells and the local environment. The most detrimental effect of a stable plaque could be ischemia of a distal organ due to lumen occlusion. In contrast, an unstable plaque with its risk of rupture and resulting thrombo-embolism will most definitely result in life-threatening cardiovascular events such as infarction or stroke.

As discussed above, fibrous cap comprises primarily of connective tissue and VSMCs, that prevents macrophage-derived tissue factor from encountering various coagulation factors in blood. Plaque instability rises from thinning of the fibrous cap due to the various cytokines, apoptosis of VSMC, and the reduction of collagen production [6,15]. Reduction of collagen production seems to be one of the major milestones in the cascade of events that leads to the rupture of the fibrous cap. Studies in mice with various impaired {collagen pathways, such as, enhanced INF-γ signaling, or genetically induced scurvy, have shown to develop plaques susceptible to rupture due to a weak fibrous cap [9]. Molecular processes such as cell adhesion, cytoskeletal restructuring, and migration play important role behind the scenes in enabling various cellular players that define the characteristics of a stable plaque. A ubiquitously expressed molecule Talin-1 aids in intercellular communications through integrin activation, and crosstalk. While the exact role of it, if any, in plaque stability needs to be determined, its expression has been shown to be downregulated in plaques vs. control arteries and has been found to exhibit a positive correlation with a stable vs. an unstable plaque. Further, miRNA-330-5p has been identified as a potential positive regulator of Talin-1 [16]. This is an example that the establishment of a plaque’s stability is multifactorial involving molecular basis as well as genetic expressions. Some studies have shown a positive correlation and a predominance of M1 subtype of macrophages in ruptured plaques; however, any causal association is under debate, and our understanding is still evolving [17].

## 4. VSMCs

Within the growing stable plaque, an interaction between the T cells and macrophages secrete a wide array of chemokines and growth factors that target circulating monocytes, local endothelial cells, and smooth muscle cells. This proliferation can lead to lesion expansion. As discussed above, the stability of the plaque is derived from the presence of a thick fibrous cap produced in part by the VSMCs. Activated VSMCs switch their phenotype from contractile to synthetic and synthesize fibrotic proteins such as collagen [18]. Typically, TGF-β is a potent activator of collagen synthesis by VSMCs; however, T-cells secrete INF-γ, which inhibits collagen synthesis, especially types I and III, that are majorly found in extracellular matrices of arteries [9,19]. It has been shown that INF-γ inhibit collagen production by VSMCs even in the presence of TGF-β [19]. The source of INF-γ is T-cells, the presence of which in atherosclerotic lesions helps to tie the link between adaptive immune response to the stability of a plaque.

VSMCs also express scavenger receptors such as LOX-1 and CD-36 that internalize ox-LDL, generating foam cells out of VSMCs, as they would with macrophages. This generation of foam cells from VSMCs is most likely due to VSMCs’ phenotypic change to resemble monocytes and mesenchymal stem cells. Endothelial and VSMCs, under the conditions of acute inflammation, start to produce pro-inflammatory molecules such as TNF-α, IL-1β, and MCP-1, among many others, which attract neutrophils to the inflammatory site. Neutrophils, in turn, act on these cells to increase their pro-inflammatory effects, thus producing a snowball effect that prolongs the inflammatory response and intimal hyperplasia [20]. Studies have shown that a knockout of the transcription factor, Klf-4, which possibly mediates the phenotypic change in VSMCs, results in a reduction of VSMC-derived macrophage-like cells, a smaller lesion size, and increased fibrous cap thickness. These changes consequently increase plaque stability [21].

## 5. Macrophages, T Cells, Platelets

Macrophages, classically have been categorized into M1, involved in pro-inflammatory pathways, and M2, participating in anti-inflammatory events. As noted above, monocytes recruited to the site of plaque formation are activated by toll-like receptors (TLRs), and INF-γ present in the micro-environment of a growing plaque to M1 subtype [17,22,23]. These M1 type macrophages go on to secrete other pro-inflammatory cytokines such as IL-1b, IL-6, TNF, IL-12, and IL-23 along with molecules such as reactive oxygen and nitrogen species that sustain the ongoing inflammation [17]. M1 type macrophage also recruit Th1 and natural killer cells to the growing plaque further exacerbating the injury due to uncontrolled inflammation [24].

While historically, atherosclerotic plaques were characterized mainly by M1 type macrophages, recent studies have brought to fore other subtypes of macrophages, such as M4, Mox, M(Hb), Mhem. These subtypes vary in their local milieu and therefore their activation, their phenotypic markers, and in their participation or role in the pathogenesis as well as the characteristics of an atherosclerotic plaque. Recent studies with mouse models and carotid plaques have shown the presence of M2 subtype in advanced lesions [25]. However, since the cause of their presence could not be narrowed down, and since macrophage phenotypic switching based on their micro-environment is well-known, such representation is challenging and studies are still ongoing to demonstrate the presence of distinct types of macrophages and their role in plaque stability and severity [17].

The reduction in collagen levels in a plaque is partly due to decreased production mediated by T cells and increased levels of breakdown by macrophages, neutrophils, endothelial cells, and SMCs. Studies have shown that macrophages secrete several matrix metalloproteinases (MMPs), such as MMP-1, MMP-2, MMP-8, MMP-9, and MMP-13, which are structurally identical to enzymes that degrade fibrillar collagen types I and III, proteoglycans, and elastin. Such MMPs also activate platelet aggregation and adhesion. Ox-LDL increases MMP-14 expression in endothelial cells, which in turn activates MMP-2, a potent gelatinase that acts on collagen IV, a constituent of the basement membrane. T-cells can bring such secretions by macrophages through the CD40 ligand (CD40L). The finding that the lack of MMPs resulted in increased and better-organized collagen in such plaques proved the role of MMPs in plaque stability. Elastases such as cathepsins S and K, and neutrophil elastase, have been found in plaques, which lower plaque stability possibly by degrading the extra-cellular matrix [19]. CD40L by T cells not only increases the secretion of MMPs but has also been attributed to the expression of tissue factor (TF) expression by macrophages. TF is the major activator of thrombosis when the cells encounter coagulation factors in blood. CD40L are also derived from platelets, which can cause its aggregation when the local environment is conducive. This can lead to local small arteries that feed the plaque to rupture leading to an intraplaque hemorrhage. This loop can continue whereby the exposed CD40L can activate TF in the local environment, leading to the growth of the thrombus, which in turn can cause further inflammation. While rupture of a plaque is the climax of the negative cardiac events, superficial erosion of the endothelial cells lining the plaque contributes to the weakening of its cap [19].

Not all small-scale changes that occur regularly within a plaque that result in mural thrombus formation lead to negative cardiac events, as seen in the thrombus formed within the vasculature of patients that did not die of heart conditions. These mural thrombi have platelets as its participants and platelet derived growth factor (PDGF) thus secreted might have led to the fibrosis of the plaque conferring it with stability. This ‘healing’ of the minor plaque ruptures lead to the ‘expansive remodeling’ of the artery rather than the ‘constrictive remodeling’ observed in an immature growing inflamed plaque [19].

## 6. Apoptosis

TNF family of cytokines from platelets has a pronounced effect on the apoptosis of VSMCs. In addition, chemokines such as IL-1beta, TNF-alpha, and IFN-gamma by activated macrophages and T cells in the immediate environment, induce apoptosis in VSMC and block collagen production [26]. Myeloperoxidase produces hypochlorous acid in the event of increased oxidative stress, which induces apoptosis in EC. Increased caspase-3 and DNA ladders in ECs support the theory that oxidative stress inherent in inflammation-induced plaque formation leads to a cyclic deterioration of plaque stability [19].

Loss of collagen through apoptosis of VSMCs, increased MMPs, and the accumulation of necrotic debris by apoptotic macrophages, all cumulatively result in an unstable plaque, increasing the chance for rupture. Risk of plaque rupture also increases with superficial erosion of endothelial monolayer, the risk of which is increased with apoptosis of endothelial cells due to inflammation [19]. Apoptosis is beneficial in clearing the cells that help eliminate oxidized elements in the initial lesion, but the same processes, if left unchecked, are detrimental during the later stages. Removing the injurious agent or resolving the inflammation could reverse the progression of the lesion from a fatty streak to an unstable plaque.

## 7. Resolution of Inflammation

Resolution of inflammation has been established as an active process that begins with and is characterized by a reduction in neutrophil recruitment and an increase in efferocytosis, a non-phlogistic clearance of cellular debris by macrophages [8].

As with the active inflammation process, its resolution involves a myriad of chemical modulators from a variety of cell populations through complex chemical pathways that are interconnected. Resolution of an ongoing inflammation is kicked off by the “class switching” of prostaglandins and leukotrienes to lipoxins, which are also derived from arachidonic acid [27].

**Figure 2:**
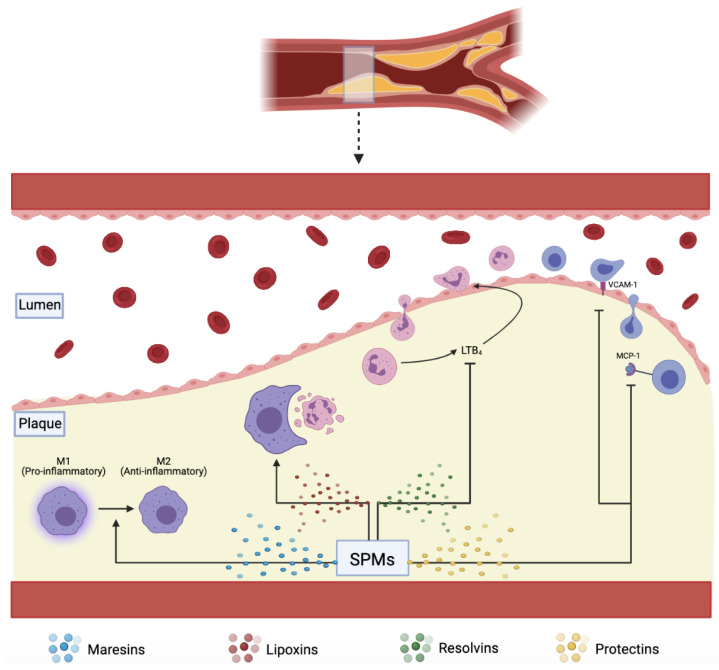
SPMs including maresins, lipoxins, resolvins, and protectins, assist in plaque resolution through various pathways: converting pro-inflammatory M1 to anti-inflammatory M2 macrophages, increasing effective efferocytosis, downregulating pro-inflammatory LTB4, VCAM-1, and MCP-1.

The resolution of acute inflammation was studied by analyzing the exudate from inflamed tissues and was found to be mediated by molecules derived from essential fatty acids, Eicosapentaenoic acid, and Docosahexaenoic acid (DHA). These molecules are termed SPMs and are classified into further subdivisions—resolvins (E and D series), protectins, lipoxins, and maresins. E-series resolvins are generated from EPA, lipoxins from arachidonic acid, while the rest, D-series resolvins, protectins, and Maresins, are derived from DHA [21,28]. These SPMs assist in reducing inflammation via several mechanisms, including increasing efferocytosis, as shown in (Figure 2) [29].

Resolution of an ongoing inflammation stimulates the increased formation of lipoxins, which facilitate resolution by stopping further recruitment of neutrophils, inducing non-phlogistic migration, and induction of macrophages to clear apoptotic neutrophils [27].

## 8. Efferocytosis

The process in which apoptotic cells are cleared is called efferocytosis. While mainly managed by macrophages, vascular smooth muscle cells and neighboring cells may also have efferocytotic roles. As discussed above, apoptosis is a significant phenomenon that defines the progression of a plaque: EC, VSMC, and foamy macrophages all undergo apoptosis fueled by the growing plaque’s chemokine environment. Tabas et al. have concluded that clearance of such apoptotic cells is the real issue within an atherosclerotic plaque than apoptosis itself [10]. Martinet et al. have shown that efferocytosis is reduced by approximately 20-fold in a plaque relative to normal [30,31]. Interestingly, macrophages that become foam cells do not unload the engulfed ox-LDL to HDLs due to their defective efferocytosis, suppressing reverse cholesterol pathways’ normal functioning. Defective efferocytosis also triggers macrophages to secrete pro-inflammatory signals such as TGF-β or IL-10. When efferocytosis is not complete, macrophages undergo cell membrane lysis spilling out necrotic chemicals such as proteases, thrombogenic tissue factors, and angiogenesis-promoting cytokines, creating more pro-inflammatory pathways (Figure 2). It is also thought that phenotype switching of macrophages from anti-inflammatory M2 to pro-inflammatory M1 has a role in diminishing the efficiency of efferocytosis [32].

**Figure 3:**
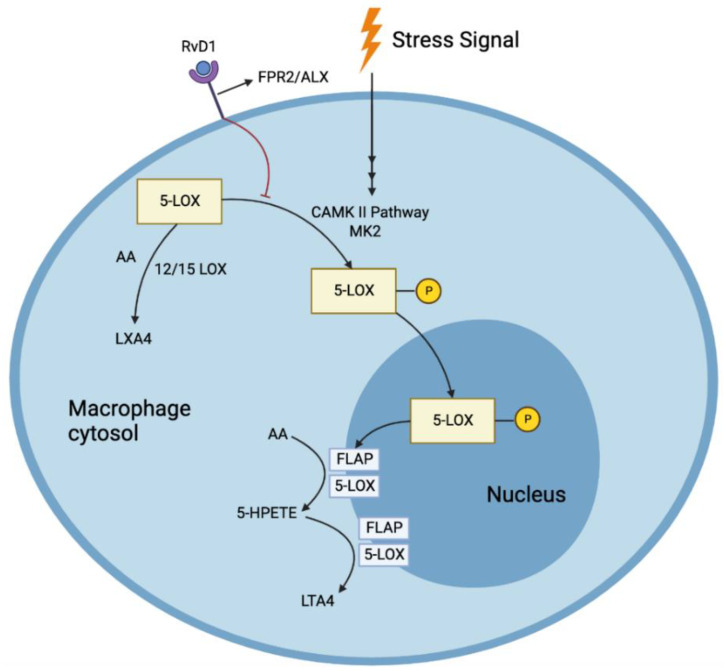
RvD1 prevents the nuclear location of 5-LOX, increasing the production of pro-resolving LXA4 and reducing the production of pro-inflammatory LTA4 in response to stress signals.

Several classes of cellular molecules highly regulate efferocytosis: “find me” ligands that recruit phagocytes to the site of apoptosis, bridging molecules that link phagocytes to their targets, and “eat me” ligands on the apoptotic cell surface. These “eat me” ligands on cell surfaces bind and activate engulfment receptors on phagocytes. A counter molecule class called “don’t eat me” ligand is present on viable cells but is downregulated in apoptotic cells. Studies have shown that ox-LDL in plaques induces auto-antibody generation within macrophages and other phagocytes. These autoantibodies mask the cell-surface “eat me” ligands on the dying cell. Ox-LDL also seems to act as a competitive inhibitor of scavenger receptors, making them less efficient in clearing apoptotic cells, as shown in (Figure 3). Calreticulin (Calr) is one of the key “eat me” ligands that binds to LDL Receptor-Related Protein (LRP1) on phagocytic cells and induces engulfment. Carriers of a risk allele at chromosome 9p21 are shown to express less Calr due to an inherited defect in TGF-β signaling, resulting in a more extensive lesion formation. Plaques of such mice models (that lack one of the 9p21 candidate genes) have been shown to exhibit plaque destabilizing features. VSMCs deficient in Calr have been shown to resist phagocytosis in vitro, induce pro-inflammatory foam-cell phenotype on cocultured macrophages, and suppress reverse cholesterol transport. It was shown that an exogenous introduction of Calr reversed these effects in-vitro [32].

As mentioned above, the “don’t eat me” ligands maintain the balance of efferocytosis and protect healthy cells from being phagocytosed. CD47 is one such ligand on healthy cells that interacts with the alpha receptor on phagocytes, shutting off the efferocytotic pathways within the phagocytes. TNF-α weakens the downregulation of CD47 in atherosclerotic plaque cells and renders them resistant to efferocytosis. CD47 blocking antibodies have shown to have beneficial effects in mouse models by preventing atherosclerotic progression, regressing the necrotic core, and preventing the plaque from rupturing [32].

A few other molecules have been implicated in the failure of efferocytosis in atherosclerosis, including Milk fat globule epidermal growth factor 8 (Mfge8) and Mer receptor tyrosine kinase (Mertk). Mfge8 is a bridging molecule between αvβ3 integrin on the macrophages and externalized phosphatidyl serine on the apoptotic body [14]. This molecule seems to be expressed less in atherosclerotic plaque. In mouse models that were created with both LDLR−/−, and transplanted Mfge8−/− bone marrow had advanced atherosclerosis with larger necrotic core and systemic inflammation. It is thought that Mfge8 might also have a role in reverse cholesterol transport by binding transglutaminase 2 [32].

Mouse models with both LDLR−/−, and transplanted Mertk−/− bone marrow showed similar plaque properties to those with absent Mfge8 and LDLR. Furthermore, a mouse with a defective kinase form of Mertk resulted in more plaque necrosis than those found in ApoE−/−. Metalloproteinases, generally found in abundance in pro-inflammatory settings, cleave Mertk into a soluble inactive form. This inactive molecule provides a decoy receptor to Growth Arrest Specific 6 and leads to competitive inhibition of efferocytosis [32].

Weissman and colleagues have found that cancer cells upregulate “don’t eat me” ligands to evade phagocytosis. Antibodies and decoy molecules that inhibit such processes and restore normal phagocytosis have been developed and are under study. If such treatments prove effective, they will have immense potential in treating atherosclerosis. Similarly, antibodies to TNF-α have been shown to reduce the expression of CD47. Anti-TNF-α antibodies are used in patients with rheumatological conditions. Such patients appear to be protected from myocardial infarction or adverse cardiovascular effects. Mouse treated with a combination of anti-CD47 and anti-TNF-α antibodies showed a better reduction in atherosclerosis than anti-CD47 alone. Given that there is a genetic susceptibility in reducing the efficiency of efferocytosis through the example of Calr expression, a genotype-driven therapy will most benefit such individuals [32].

## 9. Resolvins

### 9.1. Resolvin D Series (RvD)

RvD1 binds to two receptors, ChemR23 and BLT1, and increases macrophage phagocytosis and PMN apoptosis, respectively. RvD1 also upregulates anti-inflammatory IL-10 and downregulates pro-inflammatory LTB4 [20]. Overexpression of the enzyme 15-Lipoxygenase (15-LOX) reduced atherosclerotic plaques in rabbits [8]. Lack of resolution of inflammation and the increased ratio of LTB4 to RvD1 have been implicated as the real culprit behind what starts as a host-beneficial process to life-threatening events [33,34]. Since SPMs act in their local environments, the microclimate of the area of tissue necrosis is vital in determining their viability and action; thus, a 5-LOX closer to the cell periphery can interact with 12/15 LOX to produce RvD1 from DHA. Furthermore, RvD1 prevents the nuclear location of 5-LOX, increasing the production of pro-resolving LXA4 and reducing the production of LTA4 (Figure 3) [35]. Liquid Chromatography tandem Mass Spectrometry of human carotid plaques revealed LXA4 and RvD1 as the major SPMs, requiring 5-LOX. Furthermore, these pro-resolving molecules were much less in vulnerable plaques than the stable ones. However, the intermediates such as 5-HEPE, 15-HEPE, 17, and 14-HDHA through the actions of 5/15/12-LOX were high in vulnerable plaques, indicating that the enzymes (5-LOX and 15-LOX) themselves were bioactive even in vulnerable plaques [33]. One mechanism by which there could be a reduction in RvD1 was proposed to be the relocation of 5-LOX to the nucleus via the persistent activation of Ca^2+^/Calmodulin Dependent Protein Kinase II (CAMKII) caused by the oxidative stress from substances such as 7-ketocholesterol (7-KC) found in the atherosclerotic plaque. This was proven by the reduction in the nuclear localization of 5-LOX once CAMKII expression was suppressed in human macrophages [35]. Inhibition of CAMKII also prevented the reduction of RvD1 production by 7-KC [33]. RvD1 also blocked the synthesis of LTB4 from AA. Such pathways were mediated by the receptors formyl peptide receptor 2/ lipoxin A4 receptor (FPR2/ALX), as both a receptor blocking antibody, as well as an antagonist, blocked this reduction of LTB4 by RvD1 [35]. Nuclear localization of 5-LOX produces LTA4, which is then transformed to LTB4 through the action of LTA4 hydrolase. This nuclear localization is brought about by the phosphorylation of LOX-5 at Ser271 by p38MAPK-activated protein kinase 2 (MK2). RvD1 blocks this phosphorylation through the receptors FPR2/ALX and G-protein-coupled receptor (GPCR) Gi (and GPR32 in humans). LXA4, which shares the same receptors, also has been found to reduce the phosphorylation. RvD1 could not block the synthesis of LTB4 when incubated with large quantities of LTA4, demonstrating that RvD1 cannot alter the pathway once LTA4 is formed [35]. Fredman et al. showed that the decrease in RvD1 was associated with the progression of the atherosclerotic plaque in the aortic arch of Ldlr−/− mice fed with a western diet for 8 or 17 weeks. Analysis of the lipid mediators in early vs. late plaques showed an approximately 87-fold decrease in RvD1 in advanced plaques but no significant change in LTB4 [33]. An increase in the lesional RvD1 levels was noticed when RvD1 was administered within the physiologic range. This increase resulted in the reduction of LTB4 levels, showing that RvD1 could have facilitated this reduction by preventing the nuclear localization of 5-LOX. This simultaneous increase in RvD1 (through external administration) and reduction in LTB4 (through RvD1 mediated reduction in the nuclear localization of 5-LOX) resulted in the ratio of RvD1:LTB4 being reverted to its early plaque levels. In addition, RvD1 increased other p SPMs, reduced oxidized CAMKII, reduced oxidative stress in the plaques, enhanced efferocytosis of macrophages, reduced the size of necrotic cores, and reduced the levels of collagenase and MMP9 without a concomitant reduction in the number of macrophages or VSMCs, resulting in a thickened fibrous cap. All these effects contribute to the stability of the plaque and in slowing its progression to an advanced type [33]. RvD1 was shown to act on human PMN, in-vitro, through a GPCR receptor that was inhibited by Pertussis toxin (PTX) and reduced their actin polymerization. They also blocked β2 integrin molecules on human PMN that were regulated by LTB4. Enhancement of phagocytosis by macrophages was also observed through the interaction of the receptors ALX, GPR32, and RvD1 [36]. RvD1 was also found to limit monocyte adhesion, reactive oxygen species (ROS), and pro-inflammatory cytokine production in VSMCs derived from the saphenous vein in vitro and in rabbit arteries that underwent balloon angioplasty [20]. It was found to alter the cytoskeletal properties of arterial smooth muscle cells (ASMC) in rats, thereby inhibiting their migration. Furthermore, it reduces their proliferation, oxidative stress, and translocation of p65, a molecule vital in NF-κB stimulation, which is implicated widely in the inflammation processes. All these beneficial effects were observed without damaging the viability of ASMCs [18]. Interestingly, a positive effect of RvD1 on reducing neutrophil infiltration comes from the analysis done by Recchiuti et al. They found that RvD1, possibly through its upregulation of certain micro RNAs (miRNAs) in humans, brought about a reduction in the resolution interval by ~4 h. These miRNAs were found to target immune-competent proteins such as the NF-κB pathway and 5-LOX (in the leukotriene pathway). By blocking these pathways, the concentration of pro-inflammatory mediators is reduced [37].

**Figure 4:**
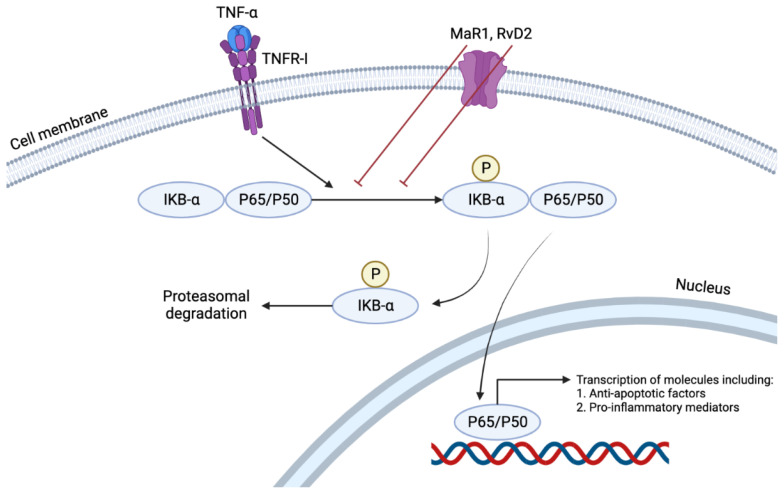
TNFα induces the NFκB pathway by nuclear translocation of p65. This pathway results in the transcription of many pro-inflammatory cytokines such as TNFα, Interleukin (IL)-1, IL-6, and IL-8. RvD2 or MaR1 treatment reduces p65 nuclear translocation, demonstrating their anti-inflammatory and pro-resolving characteristics in their local environment.

Akagi et al. demonstrated that pretreatment of ASMCsin vitro with DHA-derived SPMs, RvD2, and maresin-1(MaR1), impaired their migration towards PDGF in a dose-dependent manner by 74% and 80%, respectively at a 100 nM concentration [38]. They also showed that GPCR could have mediated this response since the reduction in migration was attenuated in the presence of PTX, which inhibits GPCR proteins [20,36,38]. Of the many immunologically active cytokines, TNFα induces the NFκB pathway by nuclear translocation of p65 (Figure 4). This pathway results in the transcription of many pro-inflammatory cytokines such as TNFα, Interleukin (IL)-1, IL-6, and IL-8. An in vitro study showed that RvD2 or MaR1 treatment of mouse ASMCs reduced p65 nuclear translocation by 24% and 28%, respectively, at a concentration of 500 nM. At the same concentration, such treatment also has reduced TNF-α induced superoxide production by 46% and 53%, respectively [38]. Furthermore, RvD2 reduced the cultured VSMCs production of VCAM-1 and ICAM-1 induced by TNF-α [20]. Such effects of these two SPMs demonstrate their anti-inflammatory and pro-resolving characteristics in their local environment. This pro-resolving action reduced the neo-intimal hyperplasia (neointima: media area ratio), which has been proven to be a result of chronic inflammation, by 67% and 71% by RvD2 and MaR1, respectively. The same study also showed a decrease in migration of neutrophils and monocytes to the area of injury, achieved by the suppression of MCP-1 expression by activated VSMCs and an increase in the M2 phenotype of macrophages which promoted resolution of the ongoing inflammation [38].

Aspirin-triggered RvD1 (AT-RvD1) has been shown to activate the nuclear factor erythropoietin 2 related factor 2 (Nrf2), increasing the expression of genes such as heme oxygenase-1 (HMOX1), and NAD(P)H quinone oxidoreductase 1 (NQO-1), aiding in combating oxidative stress in mice lung injuries [39].

### 9.2. Resovlin E Series (RvE)

Resolvin E (RvE) series (E1 and E2) molecules are produced from EPA through the action of 5-lipoxygenase (5-LOX) [40]. In addition, RvE1 receptors have been found to be GPCRs [41].

RvE series mediate the resolution of inflammation through the following means:

1. Reduction in chemotaxis of PMNs by affecting changes in their actin polymerization [42].
2. Increase in non-phlogistic phagocytic activity of macrophages [42].
3. Dose-dependent increase in IL-10 (an anti-inflammatory cytokine) by macrophages [42].
4. Downregulation of leukocyte integrin activation, reducing their response to platelet activation factor (PAF), a potent pro-inflammatory cytokine. This downregulation was postulated to be mediated through the interaction of RvE2 and leukotriene B4 receptor, BLT1. RvE1 binds BLT1 and ChemR23 equally, but RvE2 is a weak agonist of the ChemR23 receptor [42]. Platelet aggregation is one of the hallmarks of acute inflammation, brought about in part by ADP, which activates other platelets and leukocytes through intracellular signaling pathways. These pathways ultimately end with activation of platelet receptor GP IIb/IIIa, and granule secretion. RvE1 has been shown to regulate an ADP-mediated pathway that results in P-selectin surface mobilization through the ChemR23 receptor [43].

These SPMs have proven to be strong local modulators of acute inflammation through these actions. One of the major risks in atherosclerosis—thrombus formation- is avoided by preventing platelet aggregation. GP IIb/IIIa is a receptor for fibrinogen that is activated by ADP within platelets. RvE1, at concentrations of ~100 nM, has reduced fibrinogen binding to platelets. The lack of complete blockage of ADP stimulation of platelets by RvE1 is beneficiary as platelet aggregation, and thrombus formation are required for hemostasis [43].

## 10. Lipoxins

Lipoxins (LXs), as mentioned above, are endogenously made eicosanoids with anti-inflammatory properties [44]. They are produced via two reactions mediated by a set of lipoxygenases (LOX) using arachidonic acid (AA) as a precursor. AA is initially acted on by LOX-12/15 to produce an intermediate that proceeds to generate LXs via LOX-5. In humans, two types of LXs are made through this pathway, LX A4 and B4 [45]. LX A4 acts as an endogenous anti-inflammatory mediator by interacting with different players in the inflammatory immune process. One of the receptors it interacts with is a G-protein coupled ALX receptor found in many tissues and cells in the body, including neutrophils, macrophages, and endothelial cells [46]. When LX A4 interacts with the ALX receptor on neutrophils, it causes a reduction in the concentration of free oxygen radicals and production of pro-inflammatory cytokines and chemokines. It also inhibits the transmigration of neutrophils through the endothelium and induces apoptosis [46]. These changes on neutrophils is beneficial as the increase in ROS by neutrophils has been shown to play a part in plaque formation and rupture [47]. In addition, LX A4 when interacting with ALX receptor on macrophages induces phagocytosis of apoptotic leukocytes, hence ameliorating inflammation and inducing resolution [46]. Interestingly, an experiment performed in animal models with LX A4 injections demonstrated inhibition of the production of pro-inflammatory cytokines such as IL-1b, IL-6, and IL-8 and reduced infiltration of neutrophils and levels of TNF-α [46].

Similarly, LX B4 as shown to be a strong mediator in resolving inflammation during atherosclerosis [47]. Kraft et al. has recently performed an experiment depicting the effect of lipoxins in healthy individuals and those with atherosclerotic disease. The results were interesting, as they depicted lipoxins having opposing effects on neutrophils between healthy individuals and those with atherosclerosis. In patient with atherosclerosis, LX B4 seemed to inhibit the oxidative burst in neutrophils; and they describe the process of neutrophil oxidative burst as being a key player in the atherosclerotic process. On the other hand, in healthy individual LX B4 was shown to increase oxidative burst in neutrophils [47].

Furthermore, another player in clot formation and atherosclerosis are CD-11b integrins, that have been shown to regulate chemotaxis of neutrophils and neutrophil-platelet aggregation [47,48]. However, in the experiment by Kraft et al., they also illustrated that LX B4 in atherosclerotic patients caused a decrease in the synthesis of CD-11b integrin; thus, further decreasing neutrophil chemotaxis and the harmful effects of neutrophils in the atherosclerotic process [47]. Although both LX A4 and B4 have some similar effects in decreasing the detrimental effects of neutrophils, it was shown that LX B4 seemed to have a more potent effect that LX A4 [47].

In a study of the temporal relationship between peptide-derived and lipid-derived resolution compounds on patients that underwent abdominal aortic aneurysm surgery by Pillai et al., pro-inflammatory and pro-resolving mediators were closely assessed both before and after surgery. Two distinct groups of profiles emerged from this study that displayed either pro-inflammatory or pro-resolving milieu after surgery. The early resolving group (Group B) was named such, as their average LXA4 levels rose steadily from just after 5 min to 72 h post-surgery, while in the late resolving group (Group A), LXA4 peaked at 5 min post-surgery and declined significantly thereafter. Group A also had overall lower levels of ATL, higher levels of TXB2, and significantly high levels of LTB4 immediately pre- and post-unclamping of the aorta. At the same time, group B showed high levels of ATL at 5 min and 6 h post-surgery, overall low levels of TXB2 with a slight increase at 72 h post-surgery, and an overall low level of LTB4 with a mild increase at 24 h timeline. While group A patients exhibited what might look like a pro-inflammatory group, all the patients survived. Although the resolution mediating molecules showed up late, they helped recover from inflammation induced by surgeries [41]. Lastly, a standard OTC medicine, aspirin (and others of its class), which performs its action by inhibiting enzymes cyclooxygenase 1/2, allows for the action of LOX enzymes to increase as both their enzyme activities act on AA, resulting in increased LXs [45].

Our body’s innate mechanisms to deal with and curtail inflammation beyond its stipulated duration, are numerous with complex interplays within the participating agents. One such mechanism involves the nuclear factor Nrf2, a basic leucine zipper transcription factor that increases the transcription of genes that code for antioxidant proteins such as HMOX1, NQO-1, superoxide dismutase (SOD), and thioredoxin (TXN) involved in the reduction of ROS. While ROS are essential for homeostasis, elevated ROS associated with atherosclerosis induce and exacerbate endothelial dysfunction. HMOX1 has been shown to reduce atherosclerosis in mouse models [39], and upregulation of Nrf2/HMOX1 protected the human endothelial cells against TNF-α activation [49].

LXA4 prevented vascular endothelial cell (EC) damage due to oxidative stress through Nrf2 and increased the production of HMOX1. The LXA4/FPR2 receptor agonist BML-111 has shown to increase Nrf2 signaling and prevent oxidative stress in autoimmune myocarditis mouse model [39].

## 11. Maresins

Maresins, macrophage mediators in resolving inflammation are made from the ω-3 fatty acid DHA. The key enzyme in the synthesis of maresins is 12-LOX and is synthesized mainly by M2 macrophages [50,51]. Maresins appear to be tightly linked with macrophage dependent cardiac tissue regeneration and act as pro-resolving mediators by augmenting the secretion of TGF-β and decreasing concentrations of IL-6 and TNF-α [52]. MaR1 synthesized by macrophages act on BLT1 and Leucine-rich repeat -containing G-protein-coupled receptor 6 (LGR6) receptors to stimulate the phenotypic transition of macrophages from pro-inflammatory M1 to pro-resolving M2 [52,53]. In addition, MaR1 acts on retinoic acid-related orphan receptor-α (RORα) and LGR6 to enhance efferocytosis and phagocytosis by stimulating phosphorylation of several proteins such as extracellular signal-regulated kinase (ERK) and cAMP responsive element-binding protein (CREB1) [50,54,55]. In a study conducted on human saphenous vein EC and VSMC in vitro, Chatterjee and colleagues found that MaR1 weakened TNF-α induced monocyte adhesion by downregulating the cell surface adhesion molecule E-selectin. However, VCAM-1 and ICAM-1 expressions remained unchanged. MaR1 also reduced ROS generation in both EC and VSMC by downregulating NADPH oxidases (NOX4, NOX1, NOX2). Because cell adhesion, and the creation of ROS, are a couple of the hallmark events in inflammation, by minimizing them, MaR1 is a SPM that stop polymorphonuclear infiltration and inhibit ROS product. Blockage of TNF-α induction was discovered to be through inhibition of I-κ Kinase (IKK) phosphorylation and, eventually, the reduction in the nuclear translocation of the p65 subunit of NF-κB as mentioned above. Phosphorylation of IKK, in turn, phosphorylates and subjects I-kappa α to proteasomal degradation, resulting in the release of p65 from its I-κ complex, which then migrates to the nucleus to act as a transcription factor. NF-κB has been well established as a key transcription factor in synthesizing many pro-inflammatory molecules that act in a paracrine way to stimulate local inflammation [56]. It has been shown that Aspirin enables the production of Aspirin-Triggered Lipoxin (ATL), a Lipoxin A4 epimer, by interacting with the receptors FPR2/ALX found on VSMCs and macrophages in atherosclerotic plaques. The presence of these receptors correlated negatively with the clinical manifestation of the disease, implying a more stable plaque, possibly through increased collagen and decreased collagenases [57]. However, MaR1 has been shown to increase collagen synthesis leading to plaque stabilization by reducing the expression of Arginase-2 (ARG2) in endothelial cells and nitric oxide synthase 2 (NOS2) in macrophages, while increasing the expression of TGF-β1 and ARG1 [52].

Similar to AT-RvD1, MaR1 has also been found to induce Nrf2, increasing cytoplasmic HMOX1, thus reducing the levels of ROS and improved pulmonary ischemia/reperfusion injury [39].

While these studies shed light on SPM (LXA4, AT-RvD1, and MaR1)-Nrf2 relationship in increasing antioxidative proteins within pulmonary physiology, there is still debate on the overall cardioprotective effects of Nrf2, with studies showing that mice with Nrf2−/−developed less atherosclerosis [58] and HMOX1 was seen highest within human plaques with characteristics of high instability [59]. Roles of Nrf2 in lipid metabolism [58], in reduction of scavenger receptor CD36 resulting in reduced foam cell formation [60], and in NOD-, LRR- and pyrin domain-containing protein 3 (NLRP3) inflammasome induction by cholesterol crystals within the atherosclerotic plaque have been attributed to these counterintuitive results of increased atherosclerosis with complete absence of Nrf2 expression [61].

## 12. Protectins

Protectins, of which PD1 is one of the most studied, are another class of SPMs, generated from DHA, through enzymatic action on an epoxide intermediate [50,62]. PD1 issynthesized by polymorphonuclear cells, macrophages, and eosinophils [63,64,65]. The effect of PD1 is also correlated with the specific stereochemistry of the molecule; it has been shown that the R-epimer of PD1 is much more effective in its anti-inflammatory properties compared to the S-epimer [62,66]. Protectins’ anti-inflammatory activities include inhibiting neutrophil migration, as well as reducing the concentrations of TNF-α and IFN-γ by acting on GPCR37 or parkin-associated endothelin receptor-like receptor (PAELR) [67,68]. Among many other cell-protective and immunoregulatory actions, it has been shown that PD1 reduces the production of pro-inflammatory cytokines, leukocyte accumulation, and T-cell migration following an ischemic injury [67,69]. It has also been shown to downregulate the expression of VCAM-1 and MCP-1 in human aortic endothelial cells [20]. PD1 acts as an anti-inflammatory agent by regulating C-C chemokine receptor type 5 (CCR5) expression on neutrophils and decreases neutrophil infiltration of tissues [70,71]. In addition, it augments phagocytosis and efferocytosis of macrophages, which in turn clear apoptotic neutrophils [71]. Interestingly, PD1 expression has been shown to increase in the first few hours of a myocardial infarct, a correlation that further suggests a possible pro-resolving role of PD1 during early stages of inflammation [50].

## 13. Therapeutic Perspective of SPMs in Atherosclerosis

A continuous evolution of our understanding of the complex pathophysiology of atherosclerosis and the emergence of the existence of SPMs pave way for individualized and targeted pharmacotherapy in the treatment of atherosclerosis. As detailed above, this involves a complete understanding of all the pathways each of these molecules participate in, the consequences of altering such events, spatially and temporally, on both near and remotely associated structures. It would also demand from the scientific community, a feasible way to replicate in-vivo microcosms, so the bioavailability, pharmokinetics, and pharmacodynamics behave as observed and anticipated in the numerous studies leading up to it [50]. One challenge for example is the rapid metabolic inactivation of in vivo LXA4 and LXB4 by prostaglandin dehydrogenase [72].

For instance, with the knowledge of FPR2 receptor as a master switch in promoting resolution leading the cascade of events that blocks the phosphorylation and nuclear colocalization of 5-LOX, resulting in attenuation of pro-inflammatory cytokines, has led to the development of the molecule BMS986235, a FPR2 agonist by Bristol-Meyers Squibb. They had recently concluded a Phase1 trial. FPR2 has been shown to be a receptor to both LXA4, and another anti-inflammatory protein, annexin A1 (AnxA1), the interaction that leads to recruitment and polarization of macrophages to M2 phenotype. It would be interesting to watch whether the agonists of FPR2 deliver the same results as reducing pro-inflammatory molecules, and in stimulating macrophages to switch to their anti-inflammatory, M2 phenotype [73]. Another potent FPR1 and FPR2 agonist, called Compound 43 was developed by Bristol Myers Squibb. Compound 43 has been shown to induce phagocytic and chemotactic activities in mouse models, and later was patented to treat myocardial infarction [74]. Compound 17b, another agonist of FRP1/FPR2 had a similar effect on myocardial injury in mice models [75].

Another receptor involved in resolvin pathways, ChemR23, could be exploited to induce cascades leading to pro-resolution by utilizing its agonists. chemerin-9 is one such agonist, an adipokine highly expressed in white adipose tissue. Infusion of chemerin-9 resulted in decreased concentrations of TNF-α, and size of atherosclerotic lesions, and improved vascular functions [76].

G. Bannenberg et al., have shown that stable 3-oxa-ATL analogs that were resistant to β-oxidation (ZK-142/ZK-996) or the corresponding trienyne analog (ZK-990/ZK-994) exhibited anti-inflammatory effects in terms of inhibiting leukocytes and myeloperoxidase activity following oral, intravenous, or topical administration [77].

In a study by Tang et al., involving mouse models, a metabolically stable analog of aspirin-triggered resolvin D1, termed p-RvD1 (17R-hydroxy-19-para-fluorophenoxy-resolvin D1 methyl ester) has been shown to reduce damage to vascular endothelial cells resulting in markedly reduced vascular permeability in lung injury. Benzo-RvD1 (BRvD), a synthetic analog of resolvin, 17R-RvD1, reduced the migration of VSMC and inhibited NF-κB translocation in cytokine stimulated endothelial cells by 12% to 21% in a model of rat carotid angioplasty [78].

Construction of novel nano particles derived from neutrophil-derived endogenous microparticles, opens a promising door into the stable delivery of any SPM analogues. Study using these nanoparticles enriched with aspirin-triggered resolvin D1 or a LXA4 analog reduced neutrophil influx, shortened resolution timelines, and demonstrated pro-resolving actions in murine peritonitis [79].

The enzyme 5-LOX converts AA to LTA4 through the intermediates 5-hydroperoxyeicosatetraenoic acid (5-HPETE), and 5-hydroxyeicosatetraenoic acid (5-HETE) with the help of the protein 5-Lipoxygenase Activating Protein (FLAP) [80]. If the protein FLAP is inhibited, then the “class switch” from prostaglandins and leukotrienes to lipoxins could be achieved, leading to the cascade of resolution. As such, a few FLAP inhibitors/antagonists, or molecules that interfere in the FLAP mechanisms, AZD5718, BRP-201, BRP-187 have been developed that are currently being studied to understand the complexity of their consequences in inflammation and resolution.

## 14. Conclusions

As we gain more insights into the molecular mechanisms of atherosclerosis, our understanding of its pathophysiology leading to cardiovascular disease has begun to include hitherto incompletely characterized immunoactive SPMs (Figure 1). Their complex temporal and functional interdependences seem to forge a path of resolution that begins with the start of inflammation, which opens up an entirely novel way of treating the world’s number one cause of death. While the current treatment modalities are mainly damage-control with some passive preventive measures, further exploration of this new understanding will undoubtedly lead to targeted, individualized medicine that has the potential to be both preventative and curative.

## 15. Limitations of the Study

As depicted in this review, SPMs each play their role in an intertwined manner to resolve inflammation and thus curtail the pathogenesis of atherosclerosis in its early stages. Through this article we offer a simple introduction and a bird’s eye view of the role of SPMs in atherosclerosis and overview of their cellular and molecular mechanisms in resolution of inflammation, that has been proven to be implicated in the pathogenesis of the atherosclerotic cardiovascular disease. The exact molecular structure of each of the SPMs, detailed review of the studies that led to their discovery, an in-depth analysis of the interplay between various signal molecules, their receptors, and cell types in the pathogenesis of atherosclerosis are beyond the scope of this article. Also limited is the availability of a comprehensive list and analysis of any ongoing clinical trials with SPMs.

## Data Availability

Not applicable.
